# The Patient Health Questionnaire-9: Validation among Patients with Glaucoma

**DOI:** 10.1371/journal.pone.0101295

**Published:** 2014-07-07

**Authors:** Vijaya K. Gothwal, Deepak K. Bagga, Seelam Bharani, Rebecca Sumalini, Shailaja P. Reddy

**Affiliations:** 1 Meera and L B Deshpande Centre for Sight Enhancement, Vision Rehabilitation Centres, L V Prasad Eye Institute, Hyderabad, India; 2 Bausch and Lomb School of Optometry, L V Prasad Eye Institute, Hyderabad, India; Cardiff University, United Kingdom

## Abstract

**Background:**

Depression and anxiety are two common normal responses to a chronic disease such as glaucoma. This study analysed the measurement properties of the depression screening instrument - Patient Health Questionnaire-9 (PHQ-9) using Rasch analysis to determine if it can be used as a measure.

**Methods:**

In this hospital-based cross-sectional study, the PHQ-9 was administered to primary glaucoma adults attending a glaucoma clinic of a tertiary eye care centre, South India. All patients underwent a comprehensive clinical evaluation. Patient demographics and sub-type of glaucoma were abstracted from the medical record. Rasch analysis was used to investigate the following properties of the PHQ-9: behaviour of the response categories, measurement precision (assessed using person separation reliability, PSR; minimum recommended value 0.80), unidimensionality (assessed using item fit [0.7–1.3] and principal components analysis of residuals), and targeting.

**Results:**

198 patients (mean age ± standard deviation  = 59.83±12.34 years; 67% male) were included. The native PHQ-9 did not fit the Rasch model. The response categories showed disordered thresholds which became ordered after category reorganization. Measurement precision was below acceptable limits (0.62) and targeting was sub-optimal (−1.27 logits). Four items misfit that were deleted iteratively following which a set of five items fit the Rasch model. However measurement precision failed to improve and targeting worsened further (−1.62 logits).

**Conclusions:**

The PHQ-9, in its present form, provides suboptimal assessment of depression in patients with glaucoma in India. Therefore, there is a need to develop a new depression instrument for our glaucoma population. A superior strategy would be to use the item bank for depression but this will also need to be validated in glaucoma patients before deciding its utility.

## Introduction

Glaucoma is the among the leading causes of irreversible blindness worldwide, second only to cataract as the most common cause of blindness overall, and disproportionately affects women and Asians [1], [2]. Bilateral blindness from glaucoma is projected to increase from affecting 8.4 to 11 million individuals worldwide by 2020 [2]. Depression and anxiety have been reported as two common normal responses to a chronic disease such as glaucoma [3]–[8], and the patient's mental health may result in lower treatment adherence and persistence with treatment, which in turn puts him/her at an even greater risk for complications, including impending visual loss [9], [10]. Furthermore, factors such as progressive visual field (VF) loss, visual impairment, the need for multiple medical treatments, and surgery may all contribute to depression in glaucoma.

The prevalence of depressive symptoms has been estimated to be 10% to 12% in glaucoma patients [11], [12]. Specifically, higher prevalence (as high as 32%) has been reported in those with severe glaucoma [8]. The rates of depression in glaucoma patients have been reported to vary widely across regions from as low as 10% in America [13] to as high as 57% in Turkey [14]. However, caution needs to be exercised when interpreting such differences in rates of depression given that the studies vary by: (a) the definition of depression, (b) method used to diagnose depression, (c) population under consideration, and (d) time since diagnosis. Nonetheless, they provide important clinical information. The recognition and treatment of depression is crucial, because as noted earlier, depressive symptoms may adversely affect emotional well-being, adherence to treatment regimen, the ability to care for oneself, and the quality of life (QoL) [13], [14]. Therefore, providing glaucoma patients with appropriate interventions for their depressive symptoms is essential to improving their QoL and compliance with treatment. There are not enough conclusive studies regarding the association between depression and glaucoma and results of previous studies on this topic have been equivocal. For example, Wilson et al. [15] reported no increased prevalence of depression in 121 patients with open-angle glaucoma compared with 135 controls using questionnaires designed to detect depression. By comparison, Owsley et al. [16] and Skalicky et al. [8] found an association between depression and a visual function questionnaire score. In a recent study using population-based data from the National Health and Nutrition Examination Survey, Wang et al. reported that although they found glaucoma to be a significant predictor of depression even after adjustment for demographic factors and comorbidities, adjustment for general health status led to a lack of statistical significance in the relationship between depression and glaucoma [13].

Differences in the definition of depression are reflected in the variety of diagnostic methods used to assess depressive symptoms. Clinicians and other health care professionals in ophthalmology need tools to help them identify those patients with clinically significant symptoms of psychological distress quickly and efficiently without a lengthy psychiatric interview. Therefore, as opposed to diagnostic interviews such as the Structured Interview for the Diagnostic and Statistical Manual of Mental Disorders- IV (DSM-IV), which measure psychiatric disorders, self-report symptom scales such as the Patient-Health Questionnaire-9 (PHQ-9) have been developed that measure depression as a group of symptoms [17]. The PHQ-9 is a DSM-IV criterion-based instrument that was initially designed for use in primary care [17], [18]. Recently, it has been used, however, to assess depression in ophthalmic patients including those with glaucoma [13]. In addition, other scales such the Geriatric Depression Scale -15 (GDS-15), Centre for Epidemiologic Studies Depression Scale (CES-D) have also been used to assess depression in glaucoma patients [13].

Of all these instruments mentioned earlier, the PHQ-9 is appealing for several reasons. Firstly, its brevity; at only 9 items it is substantially shorter than other measures. Secondly, as compared to most other instruments developed to assess depression, the PHQ-9 was developed and validated for use with patients with systemic conditions. This is critical because it was examined for criterion validity in a population with high rates of physical symptoms and psychological distress. The PHQ-9 has demonstrated acceptability among non-psychiatric patients as well as among busy primary care providers [19], [20]. Thirdly, with the same nine items, one can establish provisional depressive disorder as well as grade depressive symptom severity, whereby PHQ-9 scores of 5, 10, 15, and 20 represent cut-offs for lower limits of mild, moderately severe, and severe depression, respectively [18], [21]. Finally and most important, is that the PHQ-9 consists of actual nine criteria on which the diagnosis of DSM-IV depressive disorder is based [18].

Given that PHQ-9 has been used in samples outside of primary care, such as in ophthalmology (e.g. glaucoma [13]), it is important that it provides reliable and robust measurements especially when measurements could impact treatment decisions. Like most instruments, however, the PHQ-9 was developed using traditional psychometric approach, i.e., classical test theory (CTT) which entails scoring the instrument by summing up raw scores and using a total PHQ-9 score (out of a maximum possible score of 27) as measure of depressive symptoms. However, the limitations of such a scoring approach have been widely acknowledged [22], [23]. At best, such a scoring approach results in ordinal-level data and limits the interpretation of the final score. More importantly, scores generated in this way should not be treated as interval measures and parametric statistics (as have been used in many publications to date, including in glaucoma patients [24]) are perhaps not appropriate. Nonetheless, transforming raw scores (such as log odds transformation through Rasch analysis) may make parametric statistics more appropriate [25]. Compared with CTT, the Rasch model overcomes the drawbacks of scoring and offers greater insight into the psychometric properties of an instrument. Specifically, it helps examine the functioning of rating scale categories; the validity (i.e. does the questionnaire measure what it purports to measure) of an instrument by evaluating the fit of individual items to the underlying construct (i.e. how well an individual item is in tandem with the whole group); and determining whether the items measure a unidimensional construct (i.e. all items measure a single concept) which is required to justify the summation of scores [25]. Application of Rasch models provides an opportunity to identify and subsequently reduce the potential bias that may exist when using instruments for assessing depression, such as the PHQ-9, in new cultural settings. Consequently, results from analyses of Rasch models can be used to increase the validity and utility of assessing for depression when the PHQ-9 is used in culturally diverse settings. Additionally, Rasch analysis helps improve sensitivity to change by reducing noise in measurement so has advantages for outcomes research.[26] Given the benefits offered by Rasch analysis, it has become a popular method to either improve the measurement properties of legacy instruments in health care, including ophthalmology [27]–[29], or develop instruments de novo [30], [31].

To date, there have been a few studies that have investigated the measurement properties of the PHQ-9 using Rasch analysis, for example, in patients undergoing coronary artery bypass graft surgery in health care and general population [32]–[34]. By comparison, there is only a single report of the application of Rasch analysis to the PHQ-9 in ophthalmology, albeit in a heterogeneous sample of people with vision loss (including 9 [8.7%] patients with glaucoma), and it was found to perform satisfactorily [35]. Given that glaucoma has been reported as a significant predictor of depression, it is important to evaluate the validity and utility of PHQ-9 in this cohort. Significant potential benefit will be gained to the glaucoma patient and his/her family members if depression is detected and managed. Therefore, the analysis, the purpose of this hospital-based cross-sectional study was to assess the psychometric properties of the PHQ-9 in a sample of glaucoma patients in South India using a Rasch model approach.

## Patients and Methods

### Study design and participants

Data for this study was collected as part of a larger study that investigated the impact of glaucoma on visual functioning in adults. Our study participants are described in more detail elsewhere [36]. Briefly, participants were drawn from the VST Glaucoma Centre, L V Prasad Eye Institute, Hyderabad, India. Eligible participants for the study were those who were aged 18 years or older, had primary glaucoma, understood and spoke English, Hindi or Telugu, had undergone glaucoma evaluation in the past 6 months at the glaucoma clinic and had at least 2 reliable automated VFs (using Humphrey Automated Field analyzer, 24-2 Swedish Interactive Threshold Algorithm – Standard, Carl Zeiss Meditec, Inc., Dublin, CA), one of which was performed in the past 6 months. Patient demographics and the type of glaucoma were abstracted from the medical record. The Patient Health Questionnaire-9 (henceforth PHQ-9) was administered along with a package of other questionnaires used to assess the impact of glaucoma on visual functioning to 198 patients (mean age  = 59.8 years) by trained interviewers on the day of their appointment. For purposes of this study, the responses of patients to PHQ-9 were included.

### Ethics Statement

Ethical approval of the study was obtained from the Ethics Committee for Human Research at the L V Prasad Eye Institute, Hyderabad, India and all consenting participants provided written informed consent. The study adhered to the tenets of the Declaration of Helsinki.

### Patient-Health Questionnaire-9

The PHQ-9 is a nine item depression module derived from the primary care evaluation of mental disorders (PRIME-MD, Pfizer Inc., New York, NY) tool [18]. It consists of 9 items (Table 1) and all of the items employ a four-category response scale: not at all (0), several days (1), more than half the days (2), and nearly every day (3). Higher PHQ-9 scores represent greater amounts of depression. Local language versions of PHQ-9 that were obtained using standard procedures were used.

**Table 1:** Item content of the Patient-Health Questionnaire-9.

Item No.	Item Description*
1.	Little interest or pleasure in doing things
2.	Feeling down, depressed, or hopeless
3.	Trouble falling or staying asleep, or sleeping too much
4.	Feeling tired or having little energy
5.	Poor appetite or overeating
6.	Feeling bad about yourself – or that you are a failure or have led yourself or your family down
7.	Trouble concentrating on things, such as reading the newspaper or watching television
8.	Moving or speaking so slowly that other people could have noticed or the opposite – being so fidgety or restless that you have been moving a lot more than usual
9.	Thoughts that you would be better off dead or of hurting yourself in some way

^*^Framing question for all above items – “Over the last 2 weeks, how often have you been bothered by any of the following”.

Response options for all the above items: not at all (0), several days (1), more than half the days (2), nearly every day (3).

### Psychometric Validation of the PHQ-9

Rasch analysis [37] was used to assess the psychometric properties of the PHQ-9 using the Andrich rating scale model [38] with Winsteps software (version 3.74.0) [39]. The Rasch measurement model has been described elegantly by Massof [40]. The procedures of Rasch analysis have been provided by us in detail earlier, so we present these in brief here [36], [41]. Rasch analysis focuses on the psychometric properties of the item, person, and rating scale categories. It allows estimates of level of depression expressed by the item (commonly referred to as item difficulty, i.e., how difficult the item is) and the person's level of depression (commonly referred to as person ability, i.e., the extent to which participants or persons possess the underlying latent trait [depression] being examined) to be made along postulated the latent trait, depression in the present case. Rasch analysis states that the probability of an individual's choosing a response on a particular item depends on both the person ability and item difficulty. Two values are used throughout the analysis: logit measures and fit statistics. The logit (or log-odds units) is the natural logarithm of the odds of a participant being successful at a specific task or an item being successfully carried out. Conventionally, 0 logit is ascribed to mean item difficulty. For the person category, logit measures indicate whether one person has more or less levels of depression than another (e.g., Does one person have lower levels of depression than another?); for items, logit measures indicate whether one item expresses more levels of depression than another (e.g., feeling down, depressed, or hopeless conveys higher levels of depression than trouble falling or staying asleep, or sleeping too much?).

For a good fitting model, we would expect that, for each item, participants with higher levels of depression would choose higher categories (such as 2 or 3), while those with lower levels of depression would consistently choose lower categories (such as 0 or 1). In Rasch analysis terms, this would be indicated by an ordered set of response thresholds for each of the items. If we consider the categories to lie on a scale, then threshold refers to the point of intersection between two adjacent categories where probability of either category being chosen is equal. The number of thresholds for an item is one less than the number of categories. The items in PHQ-9 have 4 categories and therefore have 3 thresholds. Thus, the first threshold for an item is the ability of participants for whom scoring 0 and1 is equally likely; then so on for second and third thresholds. The thresholds should demonstrate a monotonic (one direction) response process (i.e., 0 followed by 1 and so on) which indicates that with increasing levels of depression the probability of selecting higher category for an item would increase in an orderly fashion from least to most difficult. However disordering (for e.g. third threshold being located between first and second) can occur when participants have difficulty differentiating between categories. In such situations, reorganization of categories by combining them is often performed and the combination of categories that provides the best measurement precision is retained.

Given that the Rasch model is a probabilistic one, some amount of deviation of the scores of items can be expected. When an item does not perform as expected, the fit statistics (i.e. the infit mean-square statistic, infit MnSq or simply infit) flag unexpected behaviour of an item. The ideal value of the infit MnSq is 1.0. Items have high infit statistics when they do not measure the same construct as the other items in the set. Items with infit MnSq values between 0.7 and 1.3 were considered acceptable and values outside this range indicated that the items showed too much or too little variation in their response patterns (termed as misfitting items) and were considered for deletion [42]. Item deletion was an iterative process that commenced with removal of the most misfitting item and item fit as well as overall fit were evaluated after each such iteration [43].

Recent studies have suggested that fit statistics (described above) alone are inadequate for determining unidimensionality [44]–[46]. Therefore, principal components analysis (PCA) of the residuals was also used in combination with Rasch fit statistics to test the unidimensionality of the PHQ-9. The PCA transforms correlated items into principal components and the following rules of thumb were used to confirm unidimensionality: A high level of variance such as 60% or greater accounted for by the principal component is indicative of a low likelihood of additional component [47]. Also, if the variance explained by the principal component for the empirical data and model are comparable, it also indicates that there is a low possibility of finding additional components. The first contrast in the residuals indicates whether there are any patterns within the variance unexplained by the principal component to suggest that a second construct is being measured. We used the criterion of an eigenvalue of >2.0 for the first contrast which indicates that the contrast has the strength of at least two items (this is sufficient evidence of a second construct), as this is greater than the magnitude seen with random data [47].

The overall reliability of the PHQ-9 was estimated by examining the person separation reliability. Person separation reliability indicates the number of distinct strata of persons that can reliably be discerned by PHQ-9. The larger the PSR, the greater the number of distinct levels of functioning that can be distinguished by the questionnaire. The overall reliability is considered sufficient with a person separation value of 2.0 and a separation reliability of 0.8 [48].

The hierarchical order of the PHQ-9 items was examined using the person-item map provided by the WINSTEPS software. Such item hierarchy enables comparison of the level of depression expressed by the items with the persons' levels of depression and can be used to determine whether the items of the PHQ-9 cover the range of persons' levels of depression in the sample (i.e. reveal ceiling or floor effects). The average person measure was used to determine the extent to which the level of depression expressed by the items matched the level of depression experienced by participants. An absolute average person measure ≥0.5 logits indicates mistargeting (i.e. mismatch between the two entities) [49].

Adequate PSR (≥0.80) constituted the minimum acceptable measurement property of the Rasch model, for the PHQ-9 to be termed as a measure. If the instrument could not be re-engineered so as to improve PSR, analysis of higher psychometric properties such as PCA of residuals and differential item functioning was not performed.

## Results

### Participants

Of the 207 patients screened for eligibility, 198 (96%) completed the PHQ-9 among other questionnaires. The 9 participants who declined to participate (for logistical reasons) did not differ from those who did with respect to sociodemographic and clinical characteristics. The final sample was 67% male, 36% had at least 12 years of education, and 67% were not working. Mean age was 59.83±12.34 years (range, 20–87 years). A larger number of patients had primary open angle glaucoma (n = 94, 48%) or primary angle closure glaucoma (n = 82, 41%) as compared to other types of primary glaucoma. The sociodemographic and clinical characteristics of the 198 participants who responded to the PHQ-9 are summarized in Table 2.

**Table 2:** Sociodemographic and clinical characteristics of 198 participants with glaucoma who completed the Patient Health Questionnaire-9.

Variable	Result
Age, mean ± SD, years	59.83±12.34
Gender, Male, n ((%)	132 (67)
Duration of glaucoma, mean ± SD, years	8.06±6.82
Education, n (%)	
No formal education/Primary school	34 (17.2)
Secondary school	71 (35.8)
University	93 (47.0)
Employment status, n (%)	
Not working	132 (67)
Retired	77 (58.3)
Homemaker	49 (37.1)
Visual reasons	6 (4.5)
Economic status, n (%)^*^	
Low	23 (12)
Moderate	33 (17)
High	138 (71)
Positive family history of glaucoma, n (%)	39 (20)
Type of glaucoma	
Primary open angle glaucoma	94 (48)
Primary angle closure glaucoma	82 (41)
Juvenile open angle glaucoma	12 (6)
Normal tension glaucoma	10 (5)
Presenting visual acuity, mean ± SD, logMAR (Snellen)	
Better eye	0.15±0.18 (20/32^+2^)
Worse eye	0.74±0.86 (20/125^−2^)
Better mean deviation score, dB	
Mean ± SD	−12.03±9.35
Worse mean deviation score, dB	
Mean ± SD	−19.37±8.30
Glaucoma treatment category	
Pharmacologic therapy alone	67 (34)
Surgery alone	14 (7)
Laser alone	2 (1)
Combination therapy (medical and surgical/medical and laser/surgery and laser)	114 (58)

Note: SD, standard deviation; logMAR, logarithm of minimum angle of resolution (higher values indicate worse visual acuity); dB - decibels; ^*^data unavailable for 4 patients.

### Overall psychometric performance of the PHQ-9

The PHQ-9 data were fitted to the Rasch model and when we assessed the performance of the rating scale we found that the participants did not use the response categories as intended. The response categories were intended to cover a range of depression, whereby each category should be the most likely to be chosen for part of this range representing stepwise increase in frequency. However, this was not the case. Category 2, ‘more than half the days’ was not the most likely category to be endorsed at any level of depression. So we could either combine category 2 with 1 (‘several days’) or with 3 (‘nearly every day’). As noted in our methods, we decided to combine category 2 with 1 given the better measurement precision with this combination over the other. Thus, there was a reduction in the number of categories from 4 to 3 after category re-organization.

The measurement precision (an estimate of the spread or separation of persons in terms of strata or groups along the measurement construct) as assessed using PSR was 0.62 and targeting was −1.27 logits. Two items (Nos. 3 [Infit MnSq, 1.40] and 5 [Infit MnSq, 1.41]) misfit so commencing with the most misfiiting item, item 5 was deleted following which item 3 continued to misfit (Infit MnSq, 1.48). Subsequently, item 3 was also deleted. Following this, two more items misfit (Nos. 7 [Infit MnSq, 1.43] and 8 [Infit MnSq, 1.57]) which were also deleted iteratively. However, measurement precision failed to improve and targeting worsened further (i.e., there was a greater mismatch between the level of depression expressed by the items as compared to the level of depression experienced by the participants). Table 3 summarizes the iterations that were performed for the PHQ-9. Finally, five items remained which fit the Rasch model (Table 4).

**Table 3:** Summary of the overall performance of the Patient-Health Questionnaire-9.

Parameter	Ideal values	PHQ-9 (Versions)		
		Native version	PHQ-7	PHQ-5
Number of items	-	9	7	5
Number of misfitting items	0	2	2	0
Person separation reliability	>0.80	0.62	0.59	0.42
Mean item location	0	0	0	0
Mean person location (Targeting)	0	−1.27	−1.39	−1.62

**Table 4:** Item calibration (location) and fit statistics for the five items of the Patient Health Questionnaire.

Item No.	Item Description	Measure (logits)	Standard Error (logits)	Infit mean square statistic
1	Little interest or pleasure in doing things	0.13	0.23	0.84
2	Feeling down, depressed, or hopeless	−0.37	0.22	0.76
4	Feeling tired or having little energy	−1.13	0.21	1.27
6	Feeling bad about yourself- or that	−0.55	0.22	1.12
9	Thoughts that you would be better off dead or of hurting yourself in some way	1.91	0.30	0.93

## Discussion

To the best of the authors' knowledge, this is the first study that has analysed the psychometric properties of the PHQ-9 using Rasch analysis in glaucoma patients. Results of Rasch analysis in our sample indicated that there were a few fundamental problems with the use of PHQ-9. Firstly, the rating scale of the PHQ-9 – this required shortening from a four to a three-category scale, and this finding is similar to that reported in a previous study, albeit in a different population [35].

Secondly, the presence of a large number of misfitting items (44%). In a comprehensive review of comparison of 17 visual disability instruments, Khadka et al. demonstrated that the instruments that possessed disordered rating scales had higher number of misfitting items [50]. Given that the original rating scale of the PHQ-9 as has been proposed by its developers was dysfunctional in our patient population, the finding of misfitting items is, therefore, not surprising. The misfitting items in the PHQ-9 indicated that these were ambiguous, or were measuring some other construct, and therefore added noise (inaccuracy) to the measurement scale. Another reason for the misfitting items is perhaps related to their double-barrelled nature. Double barrelled items, for example, item 5 (misfit in present analyses), create confusion for the participants while responding as they aim to combine several items (and concepts) into one. For example, in the case of item 5 – ‘poor appetite or overeating’ combines two opposing activities into one; ‘poor appetite’ and ‘overeating’ represent two ends of the spectrum related to hunger. Despite these conflicting issues both have been combined into a single item. Going forward, it appears that rewording all the items in the PHQ-9 may help eliminate misfitting items, and also get rid of the double barrelled nature of the item in future studies. Therefore, as an example, we would suggest splitting this item (No. 5) into two constituent parts. While such a modification is likely to increase the length of the instrument, it may help improve the measurement properties of the instrument and render it useful for glaucoma patients. However, the modified PHQ thereof needs to be tested in future studies in patients with glaucoma. It is important to point out that despite the care we undertook during independent forward-backward translations of the PHQ-9 into local languages (done with the aim to maintain conceptual equivalence), it is plausible we may not have been able to convey the exact meaning in the local language for some of the depressive symptoms. For example, item 8 – ‘moving or speaking so slowly that other people may have noticed’ was not easily related to by our glaucoma patients perhaps. In addition, misfitting items may also be analyzed from a cultural perspective. Behaviours such as those with and sleeping and eating are perhaps not common manifestations of depression in an Indian context. However, it is likely that these results of psychometric performance of PHQ-9 may not be transferable to other countries where glaucoma patients may interpret the items differently or indeed view their depression differently depending on other cultural factors. Given this it is important that PHQ-9 is validated separately in the population to be tested.

Thirdly, the PHQ-9 lacked adequate measurement precision (evidenced by low PSR) in that it could not differentiate between glaucoma patients' in South India based on their depression symptoms. The PHQ-9 was only able to differentiate participants into two groups, i.e., lower versus higher severity of symptoms (i.e., less versus more symptomatic) given its low measurement precision (PSR). Such a low PSR (0.42) suggests that the user cannot have enough confidence in the item or person estimates. Using the CTT, the PHQ-9 was, however, shown to have high reliability (Cronbach's alpha  = 0.89 and 0.86) in an American primary care and Obstetrics Gynecology sample respectively.[18] In CTT, Cronbach's alpha is used as a reliability coefficient to represent the unidimensionality of an instrument. According to Cronbach [51], alpha estimates the ‘proportion of test variance attributable to common factors among the items’ so high inter-item correlations can lead to high Cronbach's alpha [52], [53]. Given this, Cronbach's alpha is extremely limited as an indicator of reliability. This limitation highlights the need to either use Rasch analysis in the development stage [54]–[56] or in the re-validation of instruments so as to gain a greater insight into instrument reliability [36], [41]. Although CTT methods have generally supported the psychometric properties of the PHQ-9 in primary care patients, such methods cannot facilitate the evaluation of whether items are equal in meaning to different populations [57]. Given that PSR is sample dependent, our finding of dysfunctional PHQ-9 will, therefore, only be applicable in similar populations. Thus, as indicated earlier, the performance of PHQ-9 should be tested in other populations. However, assuming this sample is typical of a glaucoma population seen in a tertiary eye care centre in the developing world, the chances of finding adequately performing PHQ-9 would be remote however. Low measurement precision (PSR) can occur due to several reasons including the presence of a smaller number of poorly targeted items in an instrument. Kroenke and Spitzer reported that the PHQ-9 was designed to be shorter in length to enable its ease of use in the busy setting of clinical practice [21]. Although respondent burden is reduced with fewer items [58], the undue shortening or inclusion of a smaller number of items can disrupt the psychometric properties of the instrument as has happened in the present study with the PHQ-9. The simplest way to increase measurement precision would be to add more items to increase the range of depression symptoms that impact patients with glaucoma. Greater measurement precision and less measurement error when evaluating depression outcomes offers the benefit of smaller sample sizes needed to detect significant differences between groups [59]. More importantly, such an enhancement in the psychometric property of an instrument reduces the resources and efforts needed from both clinical and outcomes researchers when designing and implementing studies [59]. Adding items is, however, the prerogative of the developers of the instrument (PHQ-9 in our case) so could not be pursued by us. Strategies such as focus group discussions involving the sample population to determine new items that can be appended to the existing list in the revised version of the PHQ-9 can be undertaken in future studies [60]. Of course, a superior approach will be creation of item banks that contains Rasch calibrated items pooled from different instruments that assess depression which can be administered to participants by a computerised algorithm (computer adaptive testing, CAT) that targets the ability of the participant according to his or her response and stops when the patient's estimated ability meets certain precision criteria [61], [62]. Such item banks have been developed for depression but haven't been tested as yet in ophthalmic conditions. The adaptive nature of CAT minimizes the number of items administered, thereby, reducing respondent burden. These strategies have been used in other areas of health care and it is about time that these are available in the ophthalmic field [63]–[67].

In conclusion, the PHQ-9 in its present form does not meet the requirements of the Rasch model and thus is unsuitable for measuring depression in patients with glaucoma in South India. Eye care professionals desiring to measure depression in glaucoma patients should be aware of this shortcoming of the PHQ-9 in this part of the world. Given these limitations it remains primarily a screening tool, properties that were not investigated by the present study. Although Rasch models have limitations and require caution in their interpretation when applied to a condition such as depression, they can provide unique insight into the psychometric properties of outcome measures in different patient groups. Despite the poor performance of the PHQ-9, a 5-item PHQ (with all well-fitting items) could be re-engineered after a couple of iterations, but the measurement precision failed to improve and reliability was low. That is, the final 5-item PHQ is yet ineffective given its inability to adequately discriminate among the levels of depression of patients with glaucoma in South India. Therefore more items are required in the PHQ-9 to improve its psychometric properties, specifically, measurement precision in our patient population. While other instruments such as the CES-D and HADS can be used instead, this will necessitate further validation studies using Rasch analysis prior to use in the glaucoma population.
